# Thin Film Heater for Removable Volatile Protecting Coatings

**DOI:** 10.1155/2013/928062

**Published:** 2013-11-13

**Authors:** Abid Karim

**Affiliations:** Iqra University, Defence View Shaheed-e-Millat Road (Ext.), Karachi 75500, Pakistan

## Abstract

Freshly coated aluminum mirrors have excellent reflectivity at far ultraviolet wavelengths. However, reflectivity rapidly degrades when the mirror surfaces are exposed to atmosphere. In order to avoid this problem, freshly coated aluminum surface can be protected by over-coating of a removable volatile protecting coating. This protecting coating can be re-evaporated by controlled heating or by some other methods when required. This type of removable coating has immediate application in UV space astronomy. The purpose of this paper is to demonstrate the feasibility of re-evaporation of removable volatile Zn protecting coating using a NiCr thin film heater without affecting the reflection properties of Al mirror surfaces.

## 1. Introduction

There is a considerable interest in the development of reflecting optical coating for space-borne experiments in UV astronomy in the wavelength region of 80 nm to 120 nm [1]. For technological reasons, however, this is a very difficult region of the spectrum to explore. The main problem is that the normal-incidence reflectors are very inefficient and at present, only fresh evaporated aluminum is the only known material in this region of electromagnetic radiation spectrum. Unparalleled reflectance of Al in far ultraviolet (FUV) region degrades when Al is exposed to normal atmosphere or even to the residual atmosphere of an ultrahigh vacuum system. Alternatively other materials such as thin films of SiC, B_4_C, and Ir are used. But they have low reflectance (20%–50%). The standard mirror technology is to use aluminum films over-coated with nonabsorbing thin protective layer of MgF_2_ or LiF (which prevent oxidation above 115 nm and 105 nm, resp.) [2, 3]. However, both materials absorb very strongly at shorter wavelengths as they become opaque below their cutoff wavelengths. Hamza et al. [4] and Méndez et al. [5] have investigated the reflection properties of Al surfaces over-coated with thin protected layers of C_60_ and C_70_ films. Although their results show the lowering of cutoff wavelength by the use of C_60_ and C_70_ over-coatings, reflectance of mirror surfaces was less than that of freshly evaporated Al mirrors. Bates [1] proposed *in situ* preparation of Al thin films mirrors within the orbiting satellite because it is well-established fact that the space environment of a geosynchronous satellite is not expected to cause serious degradation of pure Al mirror surfaces. However, there is no established technique for preparing such aluminum thin film mirror surfaces in an orbiting satellite. An alternative technique has been proposed by Burton [6]. In this proposed technique, a freshly coated pure Al mirror surface would immediately be over-coated with a thin volatile protective film in a ground-based laboratory to avoid oxidation of the aluminum mirror. After the launch of satellite, the volatile protecting film would be removed using a suitable technique including ion bombardment, controlled heating, and plasma etching to obtain the clean and original Al mirror surface. Controlled heating can simply be carried out by including a resistive thin film (i.e., thin film heater) below the composite coated surface (a surface coated with Al mirror and volatile protective coating of Zn). The concept of re-evaporation of volatile protecting coating in the geostationary orbital space environment is described as REVAP coating. In this paper, NiCr thin film heater for re-evaporation of Zn protecting coating is designed. Results show the feasibility of using this type of thin film heating element for REVAP coatings.

## 2. Design Considerations

### 2.1. Optimum Design of Heater

There are two commonly used configurations for the fabrication of thin film heaters. These are [7]:

- straight line track,
- meandering track.

In the case of straight line track, due to low resistance (as a result of higher width), current through the track is very difficult to control because it increases rapidly with nominal increase in the thickness of thin film [8]. Whereas, due to ease of control of current through the track and stability of film, a meandering track is found to be superior than that of straight line track for the design of a heater. Therefore, meandering track design was used as an optimum design for thin film heater.

### 2.2. Material for Heater

Throughout the work reported in this paper, NiCr has been used for the fabrication of thin film heater on top of a circular glass substrate since NiCr is a widely used material for the fabrication of thin film resistor to be used at high temperatures with high reliability [9]. Due to poor contact making ability of NiCr, gold was deposited at both ends of a track for electrical contacts when and wherever necessary.

### 2.3. Selection of Material for REVAP Protecting Coating

The requirements for protecting coating materials are as follows.

1. It should be able to protect and not to degrade the reflectivity of clean Al mirror surfaces.
2. It should have low evaporating and re-evaporating temperatures.
3. It should be thick enough to stop penetration of water vapors, oxygen, and other possible contaminants through the film and reacting with Al.
4. It has to be stable.
5. Its growth should be smooth without pinhole to avoid oxidation of Al.

All these conditions are difficult to meet. However, various inorganic compounds, metals, and some organic substances are the possible targets. A material that either has reasonably high reflectivity or has good transparency in the required spectral region can only be considered for protecting coating because these properties can be used for *in situ* alignment purposes. These criteria suggest that cadmium and zinc are the suitable materials. Due to toxic properties of Cd, Zn was selected as the material for REVAP protecting coating for the work presented in this paper. 

### 2.4. Thickness Measurement

All film layers were grown by evaporation of the required material at very high vacuum (10^−5^ torr or better) on a circular glass substrate. It was insured that all films were very smooth. Transmission of each film was measured *in situ* by comparing intensity of incoming radiation *I*_*I*_ and intensity of transmitted radiation, *I*_*O*_ using a detector and a white light source. Thickness *t* of the film was calculated by relating it to optical transmission, *T* via [8]:

(1) $$\begin{array}{c} T=1+\frac{A_{F}}{A_{G}}\left(e^{-\mu t}-1\right), \end{array}$$

where *A*_*F*_ is the film area. *A*_*G*_ is the area of glass substrate and *μ* can be calculated as [8]

(2) $$\begin{array}{c} \mu =-\frac{\ln T}{t}. \end{array}$$

## 3. Experimental Techniques and Results

**Figure 1:**
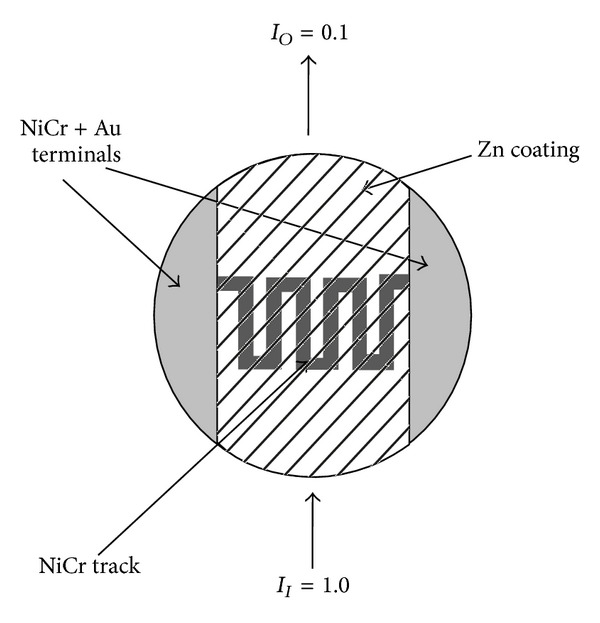
Zn coating on top of a glass plate. Meandering tack is covering a small area of glass plate.

A NiCr meandering track was deposited on a circular glass substrate. This track was covering only the central part of the circular glass substrate. Gold contacts were deposited at both ends of this track. A layer of Zn with 10% transmission was deposited on top of the NiCr meandering track in such a way that it has covered the whole substrate except the gold contact area as shown in Figure 1.

**Figure 2:**
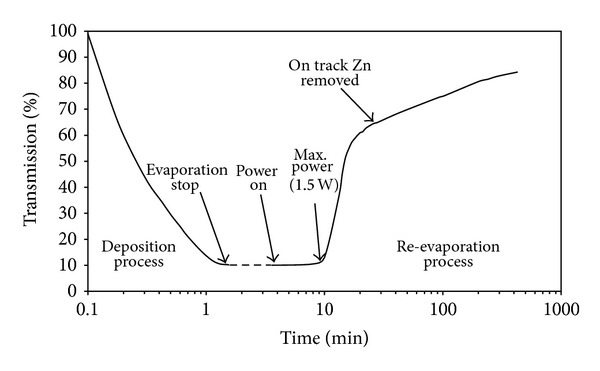
Deposition and re-evaporation process as a function of time. Meandering track is covering a small portion of Zn coated area.

After the deposition of the Zn coating on the top of NiCr meandering track, electric current was applied in a high vacuum (10^−5^ torr), so that it can be used as a heater. Current was gradually increased to a maximum value where the total power dissipation was 1.5 W. At the same time, optical transmission through the Zn coated plate was monitored continuously. As the power was increased due to increase in current through the track, temperature was increased. As the track was gradually heated up, a change in the response of detector was observed, indicating that the re-evaporation of the Zn coating was taking place. Change in optical transmission as a function of the time was watched carefully. Data obtained during the deposition and re-evaporation process is plotted in Figure 2.

**Figure 3:**
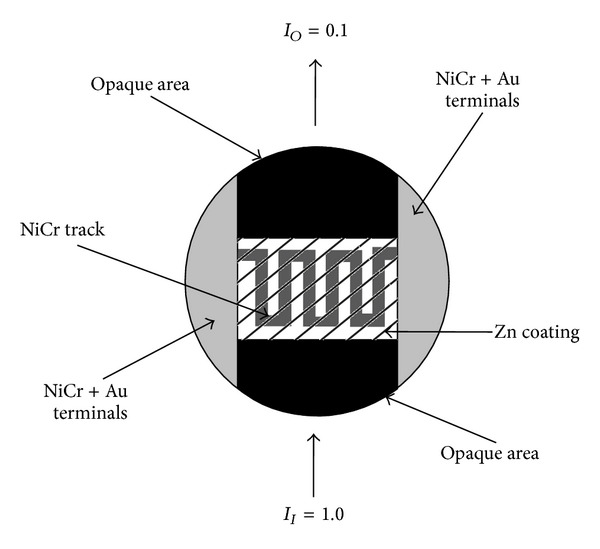
Zn coating on top of a glass plate. Meandering tack is covering a larger portion of Zn coated area.

It can be seen from Figure 2 that around 60% transmission was achieved very quickly and the maximum transmission of around 85% could only be achieved after a long time. The reason behind this behavior was that the meandering track (which was used as a heating element) was covering some area of the circular glass disc and the remaining area was without NiCr heater whereas Zn coating was deposited over the whole plate (except the gold contact areas). Zn coating close to the track re-evaporated very quickly, but the part of Zn coating which was away from the meandering track re-evaporated slowly due to temperature gradient between the glass plate and the heating track. This type of behavior suggests that the Zn could have been re-evaporated very quickly if it would have been deposited in the close vicinity of the track. Therefore, evaporation and re-evaporating process was repeated by depositing Zn coating only on the meandering track (as shown in Figure 3).

**Figure 4:**
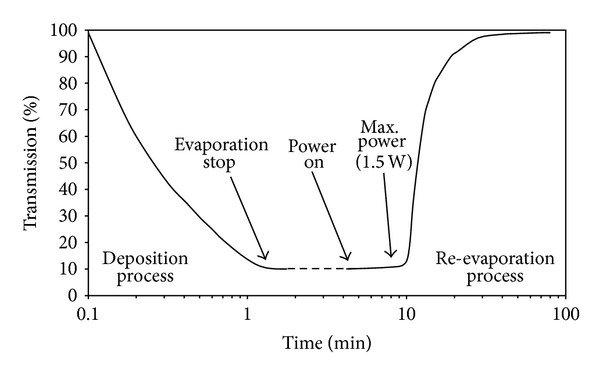
Deposition and re-evaporation process as a function of time. Meandering tack is covering a larger portion of Zn coated area.

Results obtained for this sample are plotted in Figure 4. As it can be seen from Figure 4, around 98% of transmission was achieved quickly as compared with results of Figure 2. These results ultimately suggest that the Zn coated Al mirrors should contain NiCr under layer on whole glass surface.

## 4. Conclusions

Basic experiments carried out during the work presented in this paper show the feasibility of using zinc protected aluminum mirror surfaces in space-borne UV astronomical applications. From the experimental observation, it can be concluded that in order to perform REVAP phenomena at low temperature, the vacuum should be as high as possible because the temperature required to cause re-evaporation would increase with the increase in air pressure.
